# Comparative Analysis of Genomic Island Prediction Tools

**DOI:** 10.3389/fgene.2018.00619

**Published:** 2018-12-12

**Authors:** Antonio Camilo da Silva Filho, Roberto Tadeu Raittz, Dieval Guizelini, Camilla Reginatto De Pierri, Diônata Willian Augusto, Izabella Castilhos Ribeiro dos Santos-Weiss, Jeroniza Nunes Marchaukoski

**Affiliations:** ^1^Department of Bioinformatics, Professional and Technical Education Sector, Federal University of Parana, Curitiba, Brazil; ^2^Department of Biochemistry and Molecular Biology, Federal University of Parana, Curitiba, Brazil; ^3^Department of Clinical Analysis, Federal University of Parana, Curitiba, Brazil

**Keywords:** genomic islands, pathogenic islands, mobility genes, genomic signature, virulence factors, horizontal gene transfer

## Abstract

Tools for genomic island prediction use strategies for genomic comparison analysis and sequence composition analysis. The goal of comparative analysis is to identify unique regions in the genomes of related organisms, whereas sequence composition analysis evaluates and relates the composition of specific regions with other regions in the genome. The goal of this study was to qualitatively and quantitatively evaluate extant genomic island predictors. We chose tools reported to produce significant results using sequence composition prediction, comparative genomics, and hybrid genomics methods. To maintain diversity, the tools were applied to eight complete genomes of organisms with distinct characteristics and belonging to different families. *Escherichia coli* CFT073 was used as a control and considered as the gold standard because its islands were previously curated *in vitro*. The results of predictions with the gold standard were manually curated, and the content and characteristics of each predicted island were analyzed. For other organisms, we created GenBank (GBK) files using Artemis software for each predicted island. We copied only the amino acid sequences from the coding sequence and constructed a multi-FASTA file for each predictor. We used BLASTp to compare all results and generate hits to evaluate similarities and differences among the predictions. Comparison of the results with the gold standard revealed that GIPSy produced the best results, covering ~91% of the composition and regions of the islands, followed by Alien Hunter (81%), IslandViewer (47.8%), Predict Bias (31%), GI Hunter (17%), and Zisland Explorer (16%). The tools with the best results in the analyzes of the set of organisms were the same ones that presented better performance in the tests with the gold standard.

## Introduction

Bacterial genomes have evolved and adapted over time through a variety of processes such as mutation, gene rearrangement, and horizontal gene transfer (HGT). This evolutionary pattern can be observed as increases in specific parts of sequenced genomes. In addition to genes encoding effectors of essential functions, other genes in bacterial genomes are present in many organisms, such as accessory genes acquired by HGT. The HGT process provides advantages to bacteria, enabling them to adapt to the environment (Schmidt and Hensel, 2004).

Numerous accessory genes were derived from HGT and are atypical regions known as genomic islands (GIs), which have appeared in the genomes of many bacteria. GIs play an important role in the evolution, adaptation, and diversification of bacterial genomes, carrying genes that encode proteins with diverse functions (Juhas et al., 2009). GIs were first described by Hacker et al. (1990). These researchers analyzed the genetic basis of virulence in strains of uropathogenic *Escherichia coli*. They identified gene sets containing virulence factors which were absent from commensal strains of *E. coli*. This group of genes was named as pathogenicity islands (PAI) (Hacker et al., 1990). Other studies revealed that different classes of GIs can be established depending on the biological functions of the genes within the islands. The GIs classes are: metabolic islands (MIs), which contain genes for proteins associated with metabolic properties, resistance islands (RIs), containing genes that encode proteins associated with antibiotic resistance; and symbiotic islands (SIs). This last class has various effects that depend on both the genes present and the environment. The same island can perform different functions in different environments (Hacker et al., 1997; Schmidt and Hensel, 2004).

**Figure 1:**
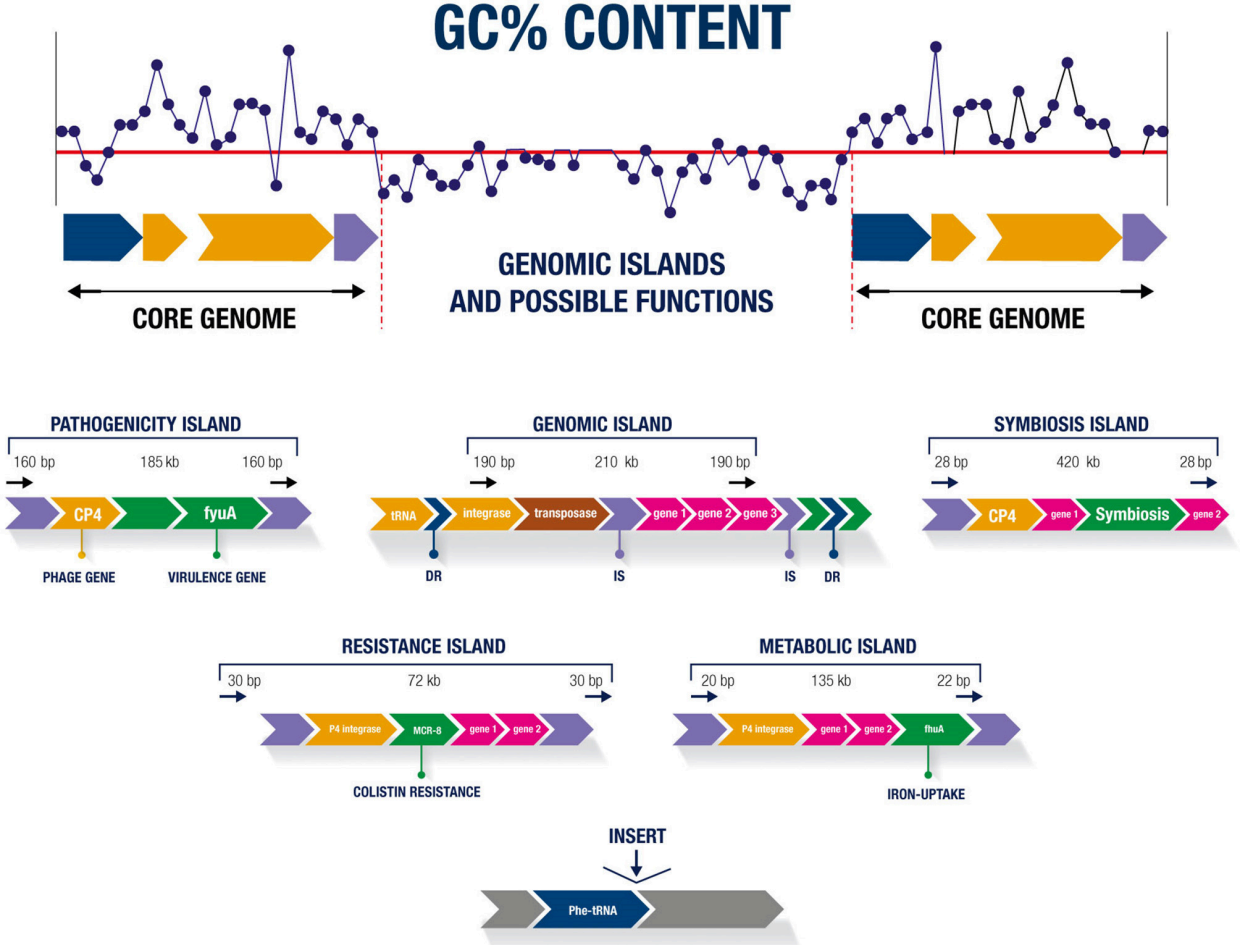
Main characteristics of genomic islands and possible functions.

Regardless of the class, most GIs have similar characteristics, such as sizes of 10–200 kb. GIs below 10 kb are known as genomic islets (Hacker and Kaper, 2000). The sequence compositions of GIs differ from that of the rest of the genome, with the specific GC% content and dinucleotide frequency the strongest indicators of their presence in the organism (Juhas et al., 2009). tRNA genes are generally found near GIs, are upstream of direct repeats (DR) sequences, and can act as target sites for enzymatic excision (Schmidt and Hensel, 2004). GIs may contain genes encoding integrins, factors involved in conjugation, and genes from phages that facilitate island transfer between organisms (Juhas et al., 2009). An insertion element (IS), integrases, and transposons related to the mobilization and deletion of genetic material may be present (Buchrieser et al., 1998; Gal-Mor and Finlay, 2006). Figure 1 shows the main characteristics of GIs and the possible functions of these sequences.

Considering the distinct properties of GIs and that they allow bacterial organisms to evolve and adapt to different environments, it is possible to understand why they spread rapidly (Juhas et al., 2007). This adaptation process is among the most important factors in generating diversity and facilitating the propagation of genes in bacteria, as the organism receives an already prepared and improved set of genes, increasing its chances of adaptation (Wilson, 2012).

The genes present in GIs are typically grouped to perform specific and advantageous functions in the bacteria. PAIs, for example, can cause major changes in the bacterial phenotype. Thus, they are the most studied GIs (Hacker and Carniel, 2001).

The ability of bacteria to transmit pathogenicity factors and antibiotic resistance factors is one of the most widely studied topics associated with GIs. The high prevalence of antibiotic resistance is an important problem facing the health care system, as it jeopardizes the success of treating infectious diseases. Changes in bacterial populations, which have increased their resistance level to various antibiotics within a few decades, show that bacteria adapt and evolve rapidly. GIs are associated with an increased distribution of virulence and antibiotic resistance factors, indicating their importance in the evolution of bacterial genomes (Juhas et al., 2009).

The large number of sequenced genomes and analyses of genetic sequences have revealed that GIs are mosaics of genes formed by HGT. Several methods for GI prediction and genomic data analysis have been developed. The main methods used by prediction tools are separated into two groups: comparative genomic analysis, whose objective is to identify variable regions in relatively close organisms (multiple genomes), and analysis of sequence composition in the organism (single genome) (Lu and Leong, 2016b).

Although numerous prediction tools are available, the accuracy of the results is insufficient. The use of only one method may not give satisfactory results; the combination of various techniques may be a better strategy for bridging the gaps in genomic island prediction (Lu and Leong, 2016b).

Recently, Bertelli et al. (2018) evaluated 20 GI predictors using a GI data set from 104 genomes (Langille et al., 2008; Dhillon et al., 2015; Bertelli et al., 2017). Analysis of the methods applied in each tool provided a broad view of the applicability of each software, revealing which predictors are better for the data set. Based on the results, (Soares et al., 2016b) and our group (Silva-Filho, 2017) have selected some previously evaluated tools according to their performance and applicability, as well as other criteria established in our lab. We investigated several predictors and selected Alien Hunter (Vernikos and Parkhill, 2006), GI Hunter (Che et al., 2014b), GIPSy (Soares et al., 2016a), IslandViewer4 (Bertelli et al., 2017), Zisland Explorer (Wei et al., 2016), and Predict Bias (Pundhir et al., 2008) for analyses. Our objective was to qualitatively and quantitatively evaluate these prediction tools against manually curated GIs. We used a set of diverse organisms and known islands curated *in vitro* to evaluate the prediction methods, island behavior in different organisms, and processes of adaptation and genomic evolution.

## Materials and Methods

### Criteria for Choosing the Prediction Tools

The predictors were chosen based on: (1) the type of analysis and method used—predictors using sequence composition, comparative, or hybrid genomics; (2) similarity/equality in pipeline construction—predictors using the same data set for tool development (such as a dataset of positive and negative GIs); predictors that integrate other tools that were previously developed and are used for the same purpose (such as GC% content and identification of tRNAs, integrases, and transposases) and predictors that determine related functions of genes present in the islands (pathogenicity, metabolism, and resistance); and (3) relevance—based on the analysis of performance and applicability in previous studies (Soares et al., 2016b; Bertelli et al., 2018) and our previous results (Silva-Filho, 2017).

#### Methodology of Analysis of GI Predictors

We chose the following predictors that use sequence composition: Alien Hunter (Vernikos and Parkhill, 2006), Predict Bias (Pundhir et al., 2008), GI Hunter (Che et al., 2014b), and Zisland Explorer (Wei et al., 2016); and the following predictors that use comparative or hybrid genomics: GIPSy (Soares et al., 2016a) and IslandViewer4 (Bertelli et al., 2017).

#### Construction of the Predictor Pipeline

##### Common data set

GI Hunter used the same dataset as IslandPick (Langille et al., 2008), developed with positive and negative GIs to create a decision tree model of the tool. GI Hunter integrates Alien Hunter into its pipeline, while IslandViewer4 integrates the IslandPick method into its predictions.

##### Integration of tools with the same purpose (GC% content)

GIPSy performs analysis using the methods incorporated in the Artemis genome visualization tool (Rutherford et al., 2000), Zisland Explorer uses GC-Profile, (Zhang et al., 2005), and Predict Bias uses the Karlin method (Karlin, 2001).

##### Integration of other tools

IslandViewer4 integrates the Islander curated database to identify tRNAs (Hudson et al., 2015) and reveals genes with characteristics of virulence, resistance, pathogenicity, and their homologous factors. GIPSy uses the HMMER3 tool (Eddy, 2011) to search the tRNAdb database (Jühling et al., 2009), identifies transposase genes using the PFAM database (Finn et al., 2010), and determines the functions of GI candidates as islands of pathogenicity, resistance, metabolism, and symbiosis. Predict Bias uses GenBank files to identify tRNAs, transposases, and integrases and determines the relationship between island function and pathogenicity.

The various methods and integrated tools used by the chosen predictors to identify the main characteristics of the GIs provides a broad view of the results for analyzing and comparing the same dataset to determine which tools give the best results. Table 1 describes the chosen tools and their main characteristics. A complete description of the prediction tools is shown in Supplementary Table 1. To evaluate these characteristics, we searched the literature for *in vitro* curated GIs that are already well-defined.

**Table 1:** Comparative characteristics of the tools.

**Predictors**	**Method**	**Seq. comp. bias^a^**	**F. of gis^b^**	**Y. of pub.^c^**
Alien Hunter	Seq. Comp.^d^	IVOM, k-mers	No	2006
GI Hunter	Seq. Comp.	IVOM, k-mers	No	2014
Predict Bias	Seq. Comp.	G+C%, codon usage, dinucleotides	Yes^e^	2008
Zisland Explorer	Seq. Comp.	G+C%, codon usage	No	2016
GIPSy	Hybrid	G+C%, codon usage	Yes^f^	2016
IslandViewer4	Hybrid	Codon usage, dinucleotides	R. G. only^g^	2009–2017

a *(Sequence composition bias)*,

b *(Genomic Islands Function)*,

c *(Year of publication)*,

d *(Sequence composition)*,

e *(Only Pathogenicity Island)*,

f *(Classifies GIs with functions of pathogenicity, resistance, metabolism, and symbiosis)*,

g *(Related genes only)*.

### Criteria for Exclusion of Prediction Tools

The predictors were excluded based on (1) low performance according to a previous study (Bertelli et al., 2018) and tools integrated by more recent versions; (2) inviable/difficulty to obtain results, such as tools with web databases but their results are offline; and (3) unsuitable installation/complex dependencies/requirements for downloading a complete external database of a very large size. The tools evaluated and excluded are shown in Supplementary Table 2.

### Criteria for Choosing the Organisms

The organisms chosen as the test set for this study were selected from those used in previous studies describing the tools; all chosen organism-genomes had been tested by at least two other tools. We tested only full genomes because not all organisms have additional information available, such as plasmids and viruses. We selected bacteria from different families to ensure diversity in our analysis. Of these bacteria, three were gram-positive and five were gram-negative (Table 2).

**Table 2:** Description of selected organisms.

**Organisms**	**Family**	**G+C% (%)**	**Gram +/–**
*Corynebacterium diphtheriae* NCTC 13129	Corynebacteriaceae	53.50	+
*Streptococcus pyogenes* M1 GAS^a^	Streptococcaceae	38.50	+
*Staphylococcus aureus* subsp*. aureus* NCTC 8325	Staphylococcaceae	32.90	+
*Escherichia coli* str. K-12 substr. MG1655	Enterobacteriaceae	50.80	**–**
*Escherichia coli* CFT073^c^	Enterobacteriaceae	50.50	**–**
*Aeromonas hydrophila* subsp. *hydrophila* ATCC 7966	Aeromonadaceae	61.50	**–**
*Pseudomonas aeruginosa* PAO1^b^	Pseudomonadaceae	66.60	**–**
*Vibriocholerae* O1 biovar El Tor N16961 chromosome I	Vibrionaceae	47.70	**–**
*Vibriocholerae* O1 biovar El Tor N16961 chromosome II	Vibrionaceae	46.90	**–**

a, b *(Organisms chosen to evaluate the processing performance time of the predictors, gram-positive and gram-negative, they represent the largest and smallest base pair contents of the entire group)*.

c *(Organism chosen as a gold standard set)*.

### Gold Standard Data Set

Using GIs previously analyzed and used as *in vitro* as reference data enabled us to evaluate the sensitivity and accuracy of the tools. The authors (Lloyd et al., 2007, 2009; Vejborg et al., 2011) identified 16 GIs in *E. coli* strain CFT073, supporting the use of this organism as the gold standard, which was used to perform manual curation of the results. Additionally, the biological composition of the GIs described and identified *in vitro* was consistent with several analytical features present in the chosen predictors.

### Analysis of the Gold Standard Data Set in the Reference Database

Several GIs of the gold standard have well-defined tRNA and PAI functions, enabling comparative analysis of the predictor results with curated databases for these specific characteristics.

For GIs with well-defined tRNAs, we used the Islander curated database (Hudson et al., 2015) for verification. IslandViewer4 implemented Islander in its last update, but only the pre-computed results are available for consultation (users uploading their own genome cannot receive Islander results). The pre-computed Islander data do not correspond to all GIs with well-defined tRNAs of the gold standard.

As most GIs identified *in vitro* function as PAIs, we consulted the Pathogenisity Island DataBase (PAI DB) (Yoon et al., 2007). In this repository, only two PAIs corresponded to the gold standard GIs.

Because of the lack of data in the reference banks when assimilated with the data from the literature, we manually curated all predictors results, identifying each biological product found by the tools and relating it to their functions and characteristics.

Table 3 shows the data from the *in vitro* curated islands of *E. coli* CFT073 obtained from previous studies (Lloyd et al., 2007, 2009; Vejborg et al., 2011).

**Table 3:** Data of GIs, PAIS, and regions with DNA of bacteriophages curated *in vitro* from the reference organism *Escherichia coli* cft073.

**GIs^a^**	**GI name**	**Locus tag^b^**	**CDS^c^**	**tRNA^d^**	**GC% content^e^ (%)**
1	GI-CFT073-leuX	c5386–c5371	15	leuX	48.15
2	PAI-CFT073-pheU	c5216–c5143	61	pheU	47.57
3	GI-CFT073-selC	c4581–c4491	70	selC	47.04
4	PAI-CFT073-pheV	c3698–c3556	124	pheV	47.08
5	PAI-CFT073-metV	c3410–c3385	25	metV	53.37
6	ϕ-CFT073-smpB	c3206–c3143	49		49.32
7	GI-CFT073-cobU	c2528–c2482	37		49.68
8	GI-CFT073-asnW	c2475–c2449	26	asnW	53.12
9	PAI-CFT073-asnT	c2436–c2418	15	asnT	58.27
10	PAI-CFT073-serU	c2416–c2392	19	serU	37.65
11	PAI-CFT073-icdA	c1601–c1518	74		50.23
12	ϕ-CFT073-ycfD	c1507–c1481	14		49.78
13	ϕ-CFT073-potB	c1475–c1400	51		50.97
14	PAI-CFT073-serX	c1293–c1165	102	serX	48.76
15	ϕ-CFT073-b0847	c0979–c0932	42		50.45
16	PAI-CFT073-aspV	c0368–c0253	83	aspV	47.43

a *(Genomic islands)*,

b *(Identifiers applied to each gene)*,

c *(Number of coding sequences)*,

d *(Transfer RNA)*,

e *(Percentage of guanine and cytosine content in the region). GI, Genomic islands; PAI, Pathogenic islands; ϕ, Islands containing predominantly bacteriophage DNA*.

### Criteria for Determining the Start and Final Position of GIs Between the Gold Standard and Tools Prediction

The gold standard GIs are represented by the first locus tag of the region and last locus tag of the region in the genome (see Table 3). We performed locus tag conversion to compare the results because the GIs predicted by the tools were identified by the initial and final position of the candidate GIs in the genome.

Because the tools used different methods, the positions of the predicted islands may not be exact compared to the positions of the standard GIs, both for the beginning and end of the island. To determine if a tool identified the gold standard island, we established a minimum of 75% coverage of the coding sequence (CDS) present in the gold standard islands. Curation was performed manually using the results from each tool.

### Criteria for Evaluating Predictor Results Between the Set of Organisms

From the results of candidate islands predicted by the different tools, we generated. GBK files for each island from Artemis software. For these files, we copied the amino acid sequence of each CDS and elaborated a multi-FASTA file separated by organism for each tool.

We used BLASTp to compared all predictor results using default parameters. From these results, we developed a set of “common islands” and “unique islands.” Because of the size variation of the predicted regions, an island may intersect two or more regions predicted by another method; in these cases, we considered the number of “Hits” between different islands.

**Figure 2:**
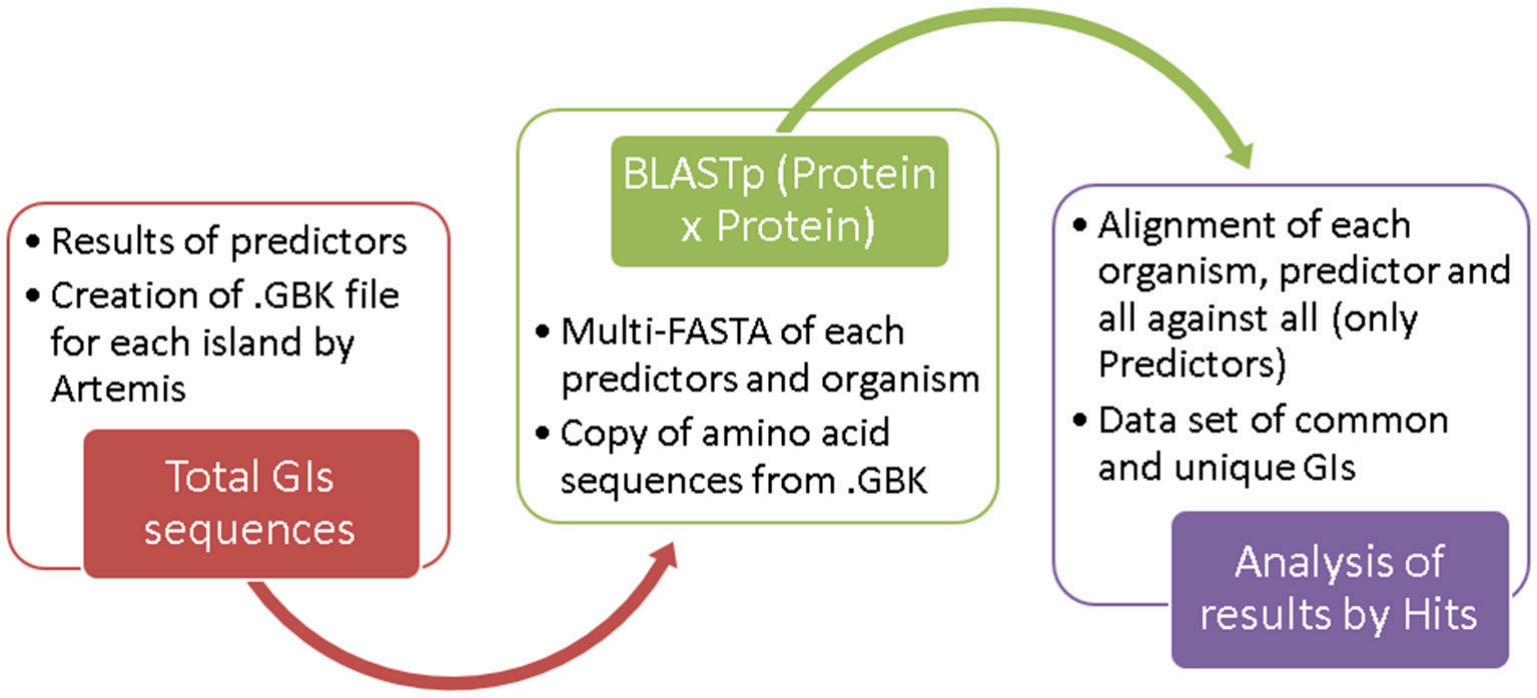
Steps in dataset creation.

We used BLASTp to align all sequences using standard parameters. Common GIs were identified by more than one predictor and showed a query coverage of 50–100%, E-value = 0.0, and identity = 100%. Unique GIs were identified by only one predictor. The flowchart in Figure 2 shows the steps used for dataset construction. The results of all BLASTp runs for the analyzed organisms are shown in Supplementary Table 3.

### Intersections Plot and Venn Diagram

The Intersection Plot Graph was developed using the UpSet tool (Lex et al., 2014), which enables investigation of data based on sets and was developed in R-language. The Venn diagrams were examined in detail using the web tool InteractiVenn (Heberle et al., 2015). The results of the Intersection Plot Graph and Venn diagram are the data sets of common and unique GIs by organism and the total data sets compared to all predictors against each other to identify similarity hits between them.

## Results

### Qualitative Analysis of GI Predictors

We analyzed the following predictive tools: Alien Hunter (Vernikos and Parkhill, 2006), GI Hunter (Che et al., 2014b), GIPSy (Soares et al., 2016a), IslandViewer4 (Bertelli et al., 2017), Zisland Explorer (Wei et al., 2016), and Predict Bias (Pundhir et al., 2008). Table 4 shows the main features of each tool and Table 5 shows the complementary information.

**Table 4:** Main features of each tool.

**Predictors**	**Platform**	**Input**	**Output**	**G. W. A^a^**	**G. R^b^**	**G. D/C^c^**
Alien Hunter	S.O^d^ Linux (Console)	.FASTA^e^	.TXT^f^/.EMBL^g^/.SCO^h^	Yes	No	No
GI Hunter	S.O Linux (Console)	.FNA^i^/.PTT^j^/.RNT^k^	.TXT/ PLOT^l^	No	No	No
GIPSy	S.O Linux/Win (GUI)^m^	.GBK^n^/.EMBL	.TXT	No	Yes	No
IslandViewer4	Web	.GBK/.EMBL	.GBK/.FASTA/.PLOT	No	No	Yes
Predict Bias	Web	.GBK	.TXT/.PLOT/HTML^o^	No	No	No
Zisland Explorer	S.O Linux/Win/Web/Mac(GUI)	.FNA/.PTT	.TXT/.PLOT	No	No	No

a *(Genome without annotation)*,

b *(Reference genome)*,

c *(Incomplete genomes drafts/contigs)*,

d *(Operating system)*,

e (Base sequences),

f *(Text file)*,

g *(Molecular Biology Laboratory)*,

h *(Score of prediction results)*,

i *(Nucleotide sequences)*,

j *(Location and attributes of proteins)*,

k *(Location and attributes of transport ribonucleic acids)*,

l *(graphical user interface)*,

m *(graphical user interface)*,

n *(GenBank genomic sequence format)*,

o *(Hypertext Markup Language)*.

**Table 5:** Additional information of each tool.

**Predictors**	**Integrated programs**	**Integrated database**
Alien Hunter	No	No
GI Hunter	IVOM^a^	No
GIPSy	SIGI-HMM^b^/HMMER3^c^/BLASTP^d^	PFAM^e^/MVIRDB^f^/ARDB^g^/CARD^h^/COG^i^/NODMUTDB^j^/tRNADB^k^
IslandViewer4	SIGI-HMM/MAUVE^l^	VFDB^m^/PATRIC^n^/VICTORS^o^/CARD
Predict Bias	No	VFDB
Zisland Explorer	GC-Profile	No

a *(Interpolated variable order motifs)*,

b *(Genomic data statistical analysis tool)*,

c *(Sequence lookup tool)*,

d *(Tool to compare sequence information in amino acids)*,

e *(Protein family database)*,

f *(Microbial database of protein, toxins, virulence factors, and antibiotic resistance genes for bio-defense applications)*,

g *(Antibiotic resistance genes database)*,

h *(Comprehensive antibiotic resistance database)*,

i *(Clusters of orthologous groups of proteins)*,

j *(database for genes and mutants involved in symbiosis)*,

k *(Transfer RNA database)*,

l *(Genome sequence alignment tool)*,

m *(Virulence factors database)*,

n *(Bacterial bioinformatics database and analysis resource)*,

o *(Pathogen-host interaction data integration and analysis system database)*.

#### Features of Each Tool

##### Alien hunter

Developed by researchers at the Sanger Institute in the UK. This software is based on Interpolated Variable Order Motifs (IVOMs), which attempt to detect atypical regions in the genome of using sequence composition analyzes such as variation of G + C content, presence of dinucleotides, and codon frequency. The predictions can be optimized using two-state Hidden Markov Models (HMM) to identify the entry point in the atypical and non-atypical regions of the genome (Vernikos and Parkhill, 2006). When the identification of these regions occurs, IVOM score is obtained, which is equivalent to how much this portion of the genome differs from the rest. Longer sequences have higher scores and more accurate predictions, whereas smaller sequences with few information have a lower score and a questionable result (Che et al., 2014a). Threshold is also established with a score, resulting from the comparison with the average of the total genome related to its similarity. Genes or genomic regions with a score below or above the threshold are possibly atypical, subsequent genes or even these atypical regions are linked to obtain candidates GIs (Lu and Leong, 2016b). Alien Hunter is able to make predictions without requiring a pre-existing annotation. Therefore, it can be used in newly sequenced genomes (Che et al., 2014a).

##### GI hunter

Developed in East Stroudsburg by the Bioinformatics Laboratory of the University of Pennsylvania. It is able to identify GIs in both bacterial and archaea genomes. It is based on analyzes of sequence composition, tRNA genes and highly expressed genes, intergenic distance, information on phages, and mobile genes (integrase and transposases), as well as the implementation of the Interpolated Variable Order Motif (IVOM) methodology that the Alien tool Hunter uses it to perform analyzes (Che et al., 2014b). In order to predict the GIs, a decision tree based prediction method with a training set was also developed. The attributes of the highly expressed genes and the intergenic distances were not explored in other tools (Che et al., 2014b).

##### GIPSy

GIPSy is an update of the Pathogenicity Island Prediction Software (PIPS), (Soares et al., 2012), developed to identify athogenic GIs in bacterial genomes. After improving, GIPSy is able to identify other candidate regions, as well as classify them according to the genes present in the GIs in relation to their biological functions (Mls, Rls, Sls). To perform the analyzes a reference genome is required. The predictions is based on the deviation of the G + C content, genomic codons, tRNA, mobility genes such as transposase, virulence factors, metabolism, symbiosis, resistance antibiotics (Soares et al., 2016a).

##### Islandviewer4

Developed at Simon Fraser University, by Brinkman Lab in Canada. It is also a database of GIs containing bacterial and archaea organisms. IslandViewer4 use three integrated methodologies: IslandPick, which uses genomic comparison, SIGI-HMM for sequence composition research and IslandPath-DIMOB, searching for atypical sequences and mobility-related genes. The interactive genome graph is provide in the web page, which gives the user a broad view of all predicted GIs with their products and features; indicates the genes related to virulence factors, pathogenicity, and antibiotic resistance. This tool does not allow the user to choose the reference genome for the IslandPick method before making the prediction. Only after receiving the results can the user choose another related genome for comparison (Bertelli et al., 2017).

##### Predict Bias

Developed in the Bioinformatics laboratory of Devi Ahila University, Indore, India. This predictor identifies genomic and pathogenic islands in prokaryotic organisms from the evaluation of sequence composition, presence of insertion elements and genes related to virulence factors. In order to predict the genes with these characteristics, an internal database was created, VFPD (A profile database of virulence factors), with the objective of searching the presence of these genes in the genome through the execution of the RPS-BLAST (Reversed Position Specific—Basic Local Alignment Search Tool) in the regions of interest. For predictions of tRNA and mobility genes such as integrases and transposases, Predict Bias use annotations of the input file GBK (Pundhir et al., 2008).

##### Zisland explorer

Developed at Tianjin University, Bioinformatics Center, in China, Zisland Explorer uses different strategies for predicting GIs. It is a non-supervised and algorithm-dependent annotation tool for automated targeting. Implements the GC + Profile software (Zhang et al., 2014), to divide the entire genome sequence into several fragments for further analysis. This approach combines homogeneity of sequences within each island and heterogeneity of sequence compositions). Zisland Explorer presents a static plot showing G + C content throughout the genome, highlighting GI candidate regions, and reporting the size and number of genes present in the candidate GIs (Wei et al., 2016).

### Performance Analysis of Island Predictors

**Figure 3:**
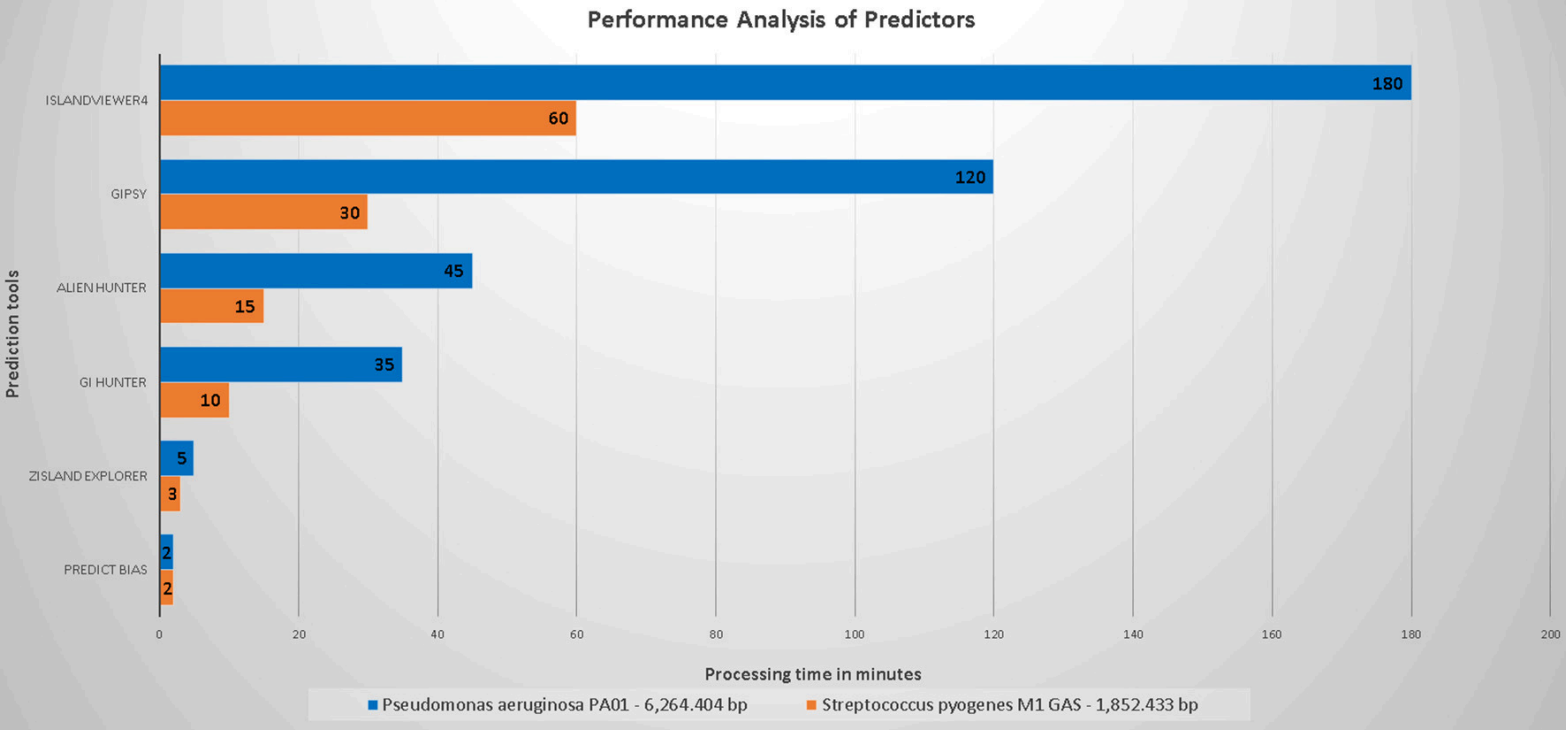
Organism with the larger genome, *Pseudomonas aeruginosa* PAO1 (6,264,404 base pairs) was compared to that with a smaller genome, *Streptococcus pyogenes* M1 GAS (1,852,433 base pairs).

Figure 3 shows the performance of the evaluated predictors with respect to the processing time.

The processing time of Predict Bias was not influenced by the genome size. Because this tool uses a set of databases, we hypothesized that some annotations had been preprocessed. The processing time of Zisland Explorer was slightly influenced by the size of the analyzed genomes. GI Hunter integrated Alien Hunter and managed to decrease the processing time difference to 25 min (70% of the time for an organism with the smallest genome) and 30 min, (65% of the time for an organism with the largest genome) compared to Alien Hunter.

Alien Hunter showed a difference in performance of 30 min (65% of the time) between the organism with the largest genome and that with the smallest genome. We did not analyze unmarked genomes. Therefore, we cannot infer an estimated time for these type of predictions. GIPSy delivered its results in 90 min (75% of the time of other genome analyses). However, this software uses two genomes for analysis (study and reference), and thus its runtime may vary.

The broadband does not appear to directly influence the time required for IslandViewer4 to perform the analyses. However, this information is not included in the published articles or on the tool page. This tool showed the highest difference in execution time, with ~120 min between the organism with the largest genome and that with the smallest genome (65% of the time). The tool uses several processes in its analyses. The time may be influenced by the number of queries being processed at a specific time in relation to queries from other organisms previously sent by other users.

In conclusion, all tools showed a relatively fast runtime, and none presented errors during execution.

### Results of Predictors Compared to the Gold Standard

**Figure 4:**
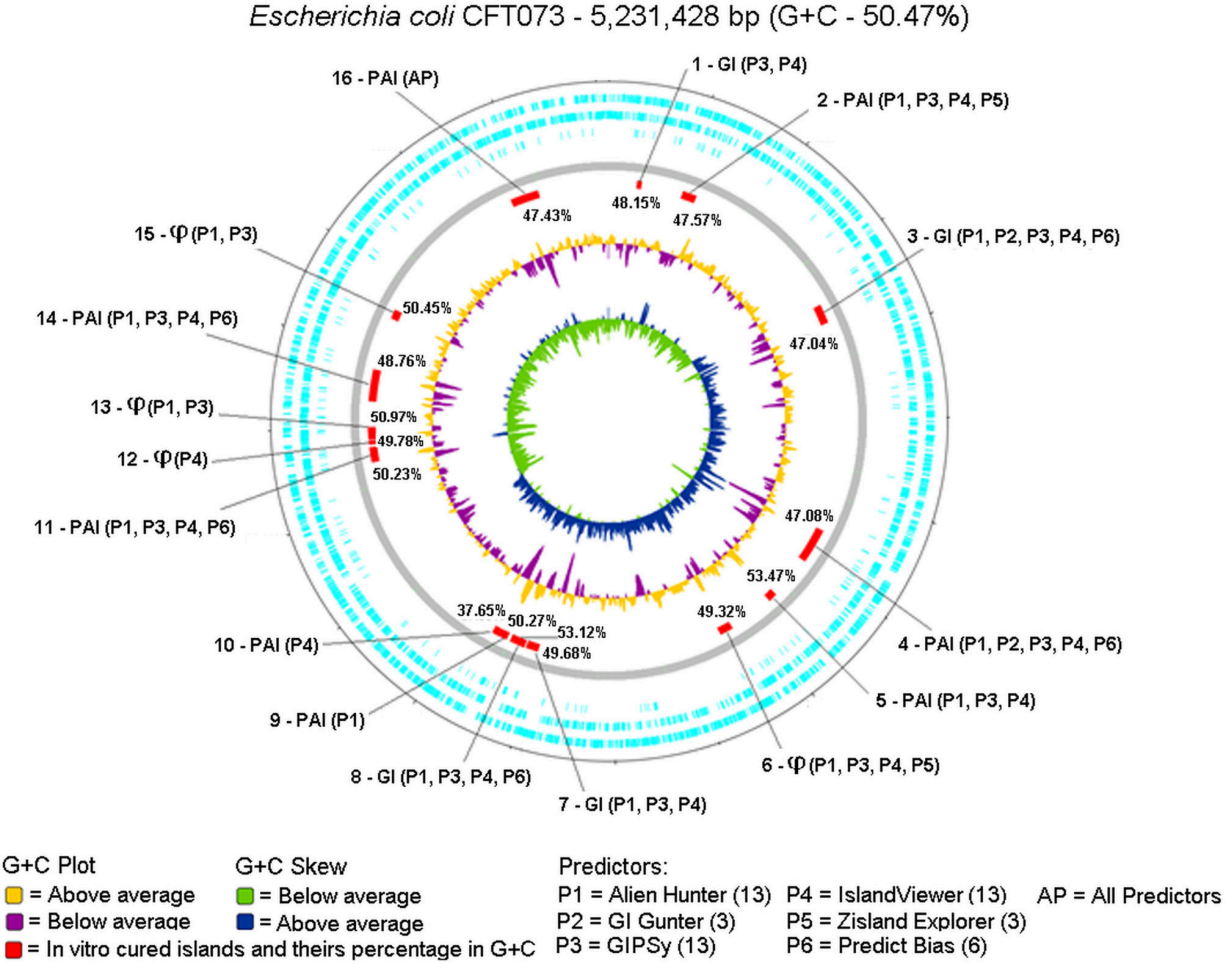
Circular genome was plotted from the Artemis tool using DNA Plotter, along with the positions of each predicted island highlighted in red, GC% content in yellow (above) and purple (below), and GC% content Skew in green (below) and blue (above). The description of each GC% content of the islands predicted together with the results of each predictor was examined. The symbol ϕ represents islands containing predominantly bacteriophage DNA.

We evaluated which tool most closely predicted the 16 GIs curated *in vitro* (Lloyd et al., 2007, 2009; Vejborg et al., 2011). Figure 4 shows the positions of the 16 *in vitro* curated GIs on the genome plotted by Artemis and the predictors used for identification.

No predictors matched the 16 GIs previously reported for the gold standard, but each island was predicted by one or more tools. Alien Hunter (P1), GIPSy (P3), and IslandViewer4 (P4) predicted the largest number of GIs, 13; GI Hunter (P2) and Zisland Explorer (P5) predicted the smallest number of GIs, three; Predict Bias (P6) identified six GIs.

Only one island was identified by all tools (GI 16). Table 6 shows a summary of the GI 16 content. This region is characterized as a PAI, containing five genes related to virulence factors: fpbABC, cdiA, picU, tosCBDA, and vat (Vejborg et al., 2011). This PAI does not contain an integrase, and 43% of the island is composed of hypothetical and non-characterized proteins.

**Table 6:** Features of gold standard gi 16 vs. predictors.

**G. standard^a^/Predictors**	**tRNA^b^**	**Transposase**	**GC% content^c^(%)**	**Vir.genes^d^**	**Hyp.prot.^e^**	**Unch. prot.^f^**	**Total CDS^g^**
**PAI**−**16**	**aspV**	**12**	**47.43**	**5**	**38**	**5**	**100**
IslandViewer4	Present	12	47.43	5	38	5	100
GIPSy	Present	12	47.38	5	38	5	99
Alien Hunter	Absent	8	46.97	4	33	5	84
Zisland Explorer	Absent	8	46.35	4	35	4	81
GI Hunter	Absent	8	46.34	3	32	4	79
Predict Bias	Absent	7	46.30	3	31	4	77

*In bold, the 16th island of the gold standard Escherichia coli CFT073*.

a *(Gold Standard)*,

b *(Transfer RNA)*,

c *(Percentage of guanine and cytosine content in the region)*,

d *(Virulence genes)*,

e *(Hypothetical proteins)*,

f *(Uncharacterized proteins)*,

g *(Coding sequences)*.

#### Features of Each Predicted Island with Manual Curation

Islands 3 and 4 were predicted by Alien Hunter (P1), GI Hunter (P2), GIPSy (P3), IslandViewer4 (P4), and Zisland Explorer (P5). The third GI had a GC% content of 47.04%, associated with the tRNA selC, and contains 2 integrases and 10 transposases. Among the five tools identified this region, GIPSy, Alien Hunter, and IslandViewer4 showed better results. GIPSy presented a GC% content of 47.29% and predicted associations with tRNA genes, integrases, and transposases. Alien Hunter and IslandViewer4 revealed a GC% content of 47.20% and associations with the tRNA and mobility genes.

The fourth island is a PAI with a GC% content of 47.08% and was associated with tRNA pheV, three integrases, and 20 transposases. Again, GIPSy, Alien Hunter, and IslandViewer4 showed the best results. GIPSy identified the region with a GC% content of 47.18%, along with all mobility genes. Alien Hunter and IslandViewer4 revealed GC% contents of 47.00 and 46.98%, respectively, but failed to predict the tRNA gene and one integrase.

The islands 2, 6, 8, 11, and 14 were predicted by Alien Hunter (P1), GIPSy (P3), IslandViewer4 (P4), Zisland Explorer (P5), and Predict Bias (P6). The second GI is a PAI with a GC% content of 47.57%, is associated with tRNA pheU, and has three integrases and 11 transposases. GIPSy and Alien Hunter showed the best results. GIPSy presented a GC% content of 47.44% and predicted an association with the tRNA gene, integrins, and transposases. Alien Hunter identified a GC% content of 47.58%, an association with the tRNA gene, and mobility genes.

The sixth GI is an island with a high content of bacteriophage DNA and GC% content of 49.32%. It is not associated with tRNA genes, and has one integrase and one transposase. Alien Hunter and IslandViewer4 showed the best predictions. Alien Hunter revealed a GC% content of 48.99% and IslandViewer4 showed 49.15%. Both tools identified the mobility genes present on the island.

The eighth GI has a GC% content of 53.12%, is associated with the tRNA asnW, and has one integrase and two transposases. GIPSy and IslandViewer4 showed the best results. GIPSy showed a GC% content of 53.03%, and IslandViewer4 gave 53.38%. Only GIPSy identified the associated tRNA and all mobility genes. IslandViewer4 failed to identify tRNAs or integrase present in the island.

The 11th island is a PAI with a GC% content of 50.23%, it is not associated with tRNA genes, but contains two integrases and four transposases. GIPSy and IslandViewer4 were the best tools. GIPSy showed a GC% content of 50.02% and IslandViewer4 gave 48.97%. GIPSy identified all mobility genes, and IslandViewer4 was associated one integrase and two transposases.

The 14th island is a PAI with a GC% content of 48.76%, is associated with tRNA serX, and contains three integrases, and 12 transposases. GIPSy, Alien Hunter, and IslandViewer4 showed the best results. The GC% content of the island predicted by GIPSy was 48.73%, by Alien Hunter 48.43% and by IslandViewer4 was 48.45%. Only GIPSy identified all associated genes, Alien Hunter and IslandViewer4 failed to identify the tRNA gene and one transposase.

Islands 5 and 7 were predicted by the tools Alien Hunter (P1), GIPSy (P3), and IslandViewer4 (P4). The fifth island is a PAI with a GC% content of 53.37%, is associated with tRNA pheV, and lacks integrases or transposases. GIPSy and Alien Hunter showed the best results. GIPSy presented a GC% content of 52.89% and is associated the pheV tRNA gene and two downstream tRNAs. Alien Hunter calculated a GC% content of 53.48% but failed to identify the tRNA gene.

The seventh island is a GI with a GC% content of 49.68%, no tRNA genes, and one integrase and seven transposases. GIPSy showed the best results. Its GC% content was 49.42%, and all genes of interest were identified.

Islands 1, 13, and 15 were predicted by the tools Alien Hunter (P1), GIPSy (P3), and IslandViewer4 (P4). The first GI has a GC% content of 48.15%, is associated with the tRNA leuX, and contains an integrase. The GIPSy tool revealed a GC% content of 46.07% and identified the tRNA gene and integrase, but its GC% content considers CDS outside the gold standard island. IslandViewer4 showed a GC% content of 48.77%, but failed to identify the tRNA, and thus the GC% content was calculated without this gene.

GI 13 contains large amounts of bacteriophage DNA and a GC% content of 50.97%. According to *in vitro* curation, this island lacks a tRNA (Lloyd et al., 2007, 2009; Vejborg et al., 2011), but in our analyses, we identified 3 tRNAs in this GI, together with 1 integrase and 1 transposase. GIPSy and Alien Hunter showed the best identification of this region. GIPSy obtained a GC% content of 51.66% and Alien Hunter showed 52.19%. Both tools identified the three tRNA genes together with the transposase but failed to identify the integrase.

The 15th GI is an island with a high content of bacteriophage DNA with GC% content of 50.45%; it has an integrase, but no tRNA genes or transposases. Two tools showed satisfactory results in this region, GIPSy and Alien Hunter. The GC% content of GIPSy was 50.28% and that of Alien Hunter was 50.47%. Both tools identified the integrase gene present on the island.

Of the 16 islands described in the gold standard, three islands (19%) were identified by only one tool: PAI 9 was identified by Alien Hunter (P1); and PAI 10 and GI 12 were identified by IslandViewer4 (P4). PAI 9 contains the fyuA gene encoding a yersiniabactin receptor, a siderophore found in pathogenic bacteria. FyuA is important for biofilm formation in disadvantageous environments with high contents of iron, such as in human urine (Hancock et al., 2008). This island has 14 CDS in total and is flanked by the tRNA gene asnT followed by an integrase. A transposase lies in the middle of the island and fyuA is at the end. Alien Hunter did not identify the tRNA gene but identified the fyuA gene. A threshold was used for identifying atypical regions in the genome; for this prediction, the threshold was 11.44 with a score of 18.24.

PAI 10 and GI 12 were only predicted by IslandViewer4 (P4). PAI 10 contains the tcpC gene, which is responsible for interfering with the innate immune response of the host (Erjavec et al., 2010). This PAI contains 26 CDS, flanked by the tRNA gene serU, and an integrase at its other end. The tcpC gene is found in the middle of the island. However, in the GBK annotation, this gene was marked as a hypothetical protein. According to The The UniProt Consortium (2017), BLAST for the tcpC gene revealed 100% identity with a Query Length of 207 and a Match Length of 307. GI 12 predominantly contains bacteriophage DNA. This island has no tRNA genes and only one integrase. IslandViewer4 identified the entire region and its CDS.

Table 7 shows the relevant products of the 16 GIs of the gold standard according to Lloyd et al. (2007, 2009) and Vejborg et al. (2011).

**Table 7:** Products of the 16 gold standard gis of *Escherichia coli* cft073 based on reference articles.

**GIs Gold standard^a^**	**tRNA^b^**	**Transposase**	**Integrase**	**GC% content^c^ (%)**	**Hyp. prot.^d^**	**Unch. prot.^e^**	**Vir. genes.^f^**	**Total CDS^g^**
1–GI	leuX	0	1	48.15	6	0	0	15
2–PAI	pheU	11	3	47.57	16	4	1	61
3–GI	selC	10	2	47.04	23	3	1	70
4–PAI	pheV	20	3	47.08	41	8	9	124
5–PAI	metV	0	0	53.37	8	1	3	25
6–ϕ:	Absent	1	1	49.32	20	5	0	49
7–GI	Absent	7	1	49.68	19	1	2	37
8–GI	asnW	2	1	53.12	4	0	1	26
9–PAI	asnT	1	1	58.27	1	0	1	15
10–PAI	serU	0	1	37.65	12	5	1	19
11–PAI	absent	4	2	50.23	23	4	1	74
12–ϕ:	Absent	0	1	49.78	9	4	0	14
13–ϕ:	Absent	1	1	50.97	28	5	0	51
14–PAI	serX	12	3	48.76	46	5	4	102
15–ϕ:	Absent	0	1	50.45	15	1	0	42
16–PAI	aspV	12	0	47.43	38	5	5	83

a *(Gold standard GIs)*,

b *(Transfer RNA)*,

c *(Percentage of guanine and cytosine content in the region)*,

d *(Hypothetical proteins)*,

e *(Uncharacterized proteins)*,

f *(Virulence genes)*,

g *(Total encoding sequences)*,

*ϕ: (Island containing predominant bacteriophage DNA)*.

*PAI, Pathogenicity island*.

### Total Results of Each Predictor

To compare the total results of each predictor, a survey of the 16 GIs in the gold standard was performed considering the main products such as tRNAs, integrases, transposases, hypothetical, and uncharacterized proteins, and the number of CDS in the region. We included all protein products in the CDS count. To account for tRNA genes, we considered that the tool identified tRNA when it was present in the GI region or when the region contained the last product before the tRNA. No tool presented exact predictions of the initial and final GI positions compared to the gold standard. Some predictions lost CDS, while others included other genetic components. To guarantee that the sum result did not affect the total gene count, any island identified by the predictors containing additional CDS or any evaluated product compared to the gold standard was excluded from the final count. Table 8 shows the total number of relevant CDS present in the 16 GIs of the gold standard compared to the total results of the predictors.

**Table 8:** Total of relevant cds present in the 16 islands described of the gold standard compared to the results of the predictors.

**G. standard^a^/Predictors**	**tRNA^b^**	**Integrase**	**Transposase**	**Hyp. prot.^c^**	**Unch prot.^d^**	**Total CDS^e^**	**Coverage %**
***Escherichia coli*** **CFT073**	**13**	**22**	**81**	**309**	**51**	**807**	**100**
GIPSy	11	16	80	276	40	738	91
Alien Hunter	5	13	70	241	37	655	81
IslandViewer4	3	12	75	239	37	636	78
Predict Bias	1	4	26	101	17	182	22
GI Hunter	0	0	15	56	11	143	17
Zisland Explorer	0	1	13	47	9	137	16

*In bold, total products present in the 16 islands of the gold standard*.

a *(Gold standard)*,

b *(Transfer RNA)*,

c (Hypothetical proteins),

d *(Uncharacterized proteins)*,

e *(Number of coding sequences)*.

We found that Predictors Bias, GI Hunter, and Zisland Explorer missed many products (625, 664, and 670 CDS, respectively). This loss affected important and characteristics genes in the GIs, such as integrases, transposases, and tRNAs. IslandViewer4 and Alien Hunter identified small numbers of tRNA genes at five and three, respectively, but their overall predictions covered a large portion of the CDS (81 and 78%, respectively). GIPSy showed good results with 91% CDS coverage and identified the largest number of products.

The data shown in Table 9 were derived from confusion matrices calculated with data from the gold standard. When the predicted islands were coincident with the 16 GIs described for the gold standard, they were classified as true-positives. The island regions of the gold standard not included among the predicted regions were classified as false-negatives, and predicted regions not present in the gold standard were classified as false-positives. The number of false-positives should be lower. Within the scope of this article, we consider that we did not used the proper methods to estimate false-negatives.

**Table 9:** Precision, recall, and f-score of 16 gold standard Gls.

	**Alien Hunter (P1)**	**GI Hunter (P2)**	**GIPSy(P3)**	**IslandViewer4 (P4)**	**Zisland Explorer (P5)**	**Predict Bias (P6)**
Precision	16%	17%	34%	17%	27%	8%
Recall	81%	19%	81%	81%	19%	38%
F-Score	0.263	0.176	0.481	0.277	0.222	0.130

*Predictors followed by the total number of correct predictions. (P1-13), (P2-3), (P3-13), (P4-13), (P5-3), and (P6-6)*.

Individually, no predictors successfully identified the 16 islands of the gold standard. Alien Hunter (P1), GIPSy (P3), and IslandViewer4 (P4) showed the best performance, achieving the same (13/16, 81%) sensitivity. In general, the tools identified many false-positives, explaining the low precision. GIPSy and Zisland Explorer showed the highest false-positive values of 34 and 27%, respectively. The F1-score correlates accuracy and sensitivity; comparison of the tools by this metric revealed that the best prediction results were those of GIPSy (0.481), IslandViewer4 (0.277), and Alien Hunter (0.263).

### Results of Total Hit Intersections Between Predictors in All Organisms

**Figure 5:**
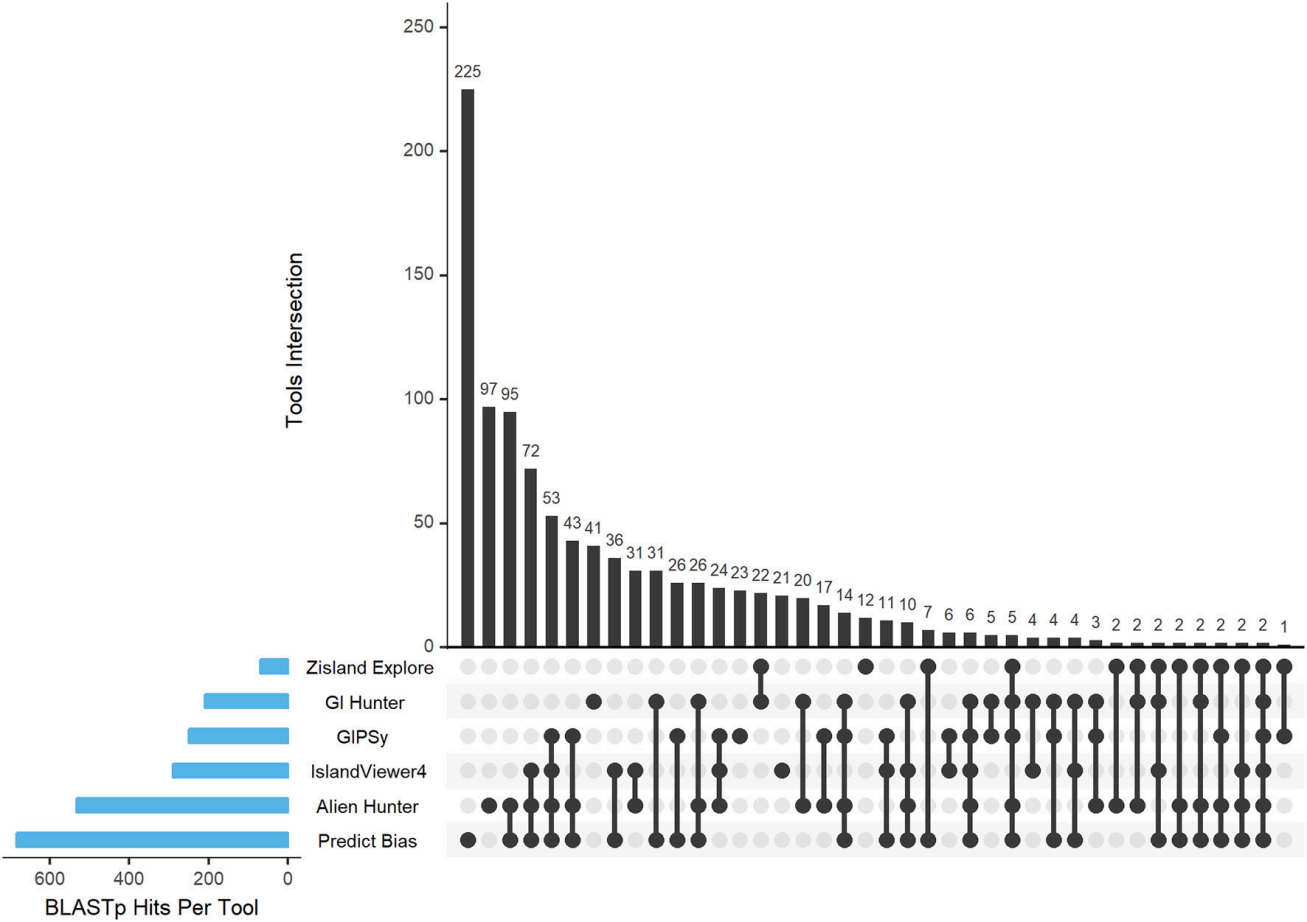
BLASTp hits of the tool intersection. The blue bars gradually display the tool intersections. The black circles show the intersections of the tools between them and the black bar graphs show how many times these intersections happened.

Based on the BLASTp data, we obtained the hits in the intersection of all results against all predictors and organisms. Figure 5 shows the Intersection Plot Graph of the tools, while the complementary Supplementary Figures 1–9 show the results for each organism on a Venn diagram. In the organism *Corynebacterium diphtheriae* NCTC 13129, all tools predicted two common GIs. In other organisms, no GIs were predicted by all tools. Additionally, more than half of the predictions made by the two tools corresponded to unique GIs. Thus, there was a tendency for a large number of false-positive results in the predictions. Table 10 shows these results together with their percentages.

**Table 10:** Hits intersections between predictors in all organisms and total number of islands predicted.

**Predictors**	**Predict Bias**	**Alien Hunter**	**IslandViewer4**	**GIPSy**	**GI Hunter**	**Zisland Explorer**
Total of hits	684	533	289	249	209	70
Total of predicted GIs	333	320	161	89	81	33
Common islands	108 (32%)	223 (70%)	140 (87%)	66 (74%)	40 (49%)	21 (64%)
Unique islands	225 (67%)	97 (30%)	21 (13%)	23 (26%)	41 (51%)	12 (36%)

*Total of hits are the result intersections between predictors; total of predicted Gls is the total GIs predicted by the tools, common islands are the total GIs predicted by two or more tools, unique islands are the total of GIs predicted by only one tool*.

Common GIs were predicted by more than one tool, and we consider that these results were true-positives. Unique GIs were predicted by only one tool, which we assumed were false-positives.

## Discussion

Current computational methods for predicting GIs have been developed to address issues related to genome sequences, which are aggravated by the vast amounts of biological data currently available. According to Langille et al. (2008), incomplete sequences resulting from metagenomic projects increase the problems faced by GI prediction approaches. This scenario can be improved by developing a tool that integrates multiple approaches, such as machine learning (Soares et al., 2016a). However, some predictors did not classify GIs with the expected efficacy.

Recent studies, such as those by Soares et al. (2016b), Lu and Leong (2016b), and Bertelli et al. (2018), evaluated various tools and their methods, explaining their advantages, disadvantages, and prediction limitations, but none used *in vitro* cured GIs already described and well-documented in the literature. Bertelli et al. (2018) compared the performance of several predictors using a data set from Langille et al. (2008), and showed that the different structures and characteristics of the GIs lead to discrepant results when predictions are made using only one method.

The criteria for choosing and how to use the tools may vary according to the characteristics of each studied genome. For example, Alien Hunter is able to perform predictions of GI candidate regions in the genome without annotation including a score for each of them, thus facilitating their identification of related products. Depending on the routine of the research laboratory, the curation of the annotation may take time and with this feature of this tool, it is possible to annotate and curate only the candidate region, helping the researcher in advance.

GI Hunter presents some different characteristics when compared to the other tools in its methodology, for example, the attributes of the highly expressed genes and the intergenic distances have not yet been explored in other tools.

GIPSy is the only tool that enables the researcher to choose the reference genome to analyze along with their study organism and determines the function of each island according to its genomic content of the GI candidate. This possibility allows many approaches in different organisms and species and may present new findings and satisfactory results.

IslandViewer4 also performs a comparative genomics approach in one of its methodologies for prediction but does not allow the research to choose its reference genome at the first moment, only after the results it is possible to make the comparison with other organisms that are deposited in the database. However, this tool is web-based and has an interactive circular genome graph, saving the researcher time and not requiring software installation requirements.

Predict Bias is also a web tool, but the output data of this tool is presented according to the locus tag of the genome, making it very difficult, and time-consuming to determine the beginning and end regions of the island in the genome.

Zisland Explorer works mainly with the G + C content. Depending on the genome characteristic and its variance of the G + C content, the researcher can perform several approaches, since it is one of the main characteristics of the GIs. This tool still works via the web, making it easier to save time for research.

In this study, we investigated several tools and their prediction characteristics to overcome some of the limitations observed in similar research during their analyses. We used an organism with *in vitro* curated GIs to verify if a unique tool/method could identify all islands. The limitations described by other authors during the predictions of GIs were also observed in this study. Even when using curated GIs described in the literature, the gap in tool predictions remained present.

In our gold standard, only one GI of the 16 curated *in vitro* was predicted by all tools. GIPSy, Alien Hunter, and IslandViewer4 showed the best overall results. GIPSy achieved 91% coverage of all CDS, followed by Alien Hunter with 81%, and IslandViewer4 with 78%. The Alien Hunter tool identified an isolated PAI and IslandViewer4 a PAI and region with dominant bacteriophage DNA. Each PAI contained virulence genes important for understanding pathogenicity factors and mechanisms that benefit the organism. Zisland Explorer, GI Hunter, and Predict Bias did not achieve satisfactory results; these tools failed to identify 84, 83, and 78%, respectively, of the characteristic genes of islands curated *in vitro*.

A characteristic of GIs curated *in vitro* is the presence of tRNAs, integrins, and transposases. GIPSy identified the largest number of these products using integrated tools, Alien Hunter and IslandViewer4 showed similar results, but tRNA identification using these two tools was low. The integration of some methods/tools may improve these predictions. The quality of the results in the gold standard predictions apply to the rest of the data set. The three tools (Alien Hunter, GIPSy, and IslandViewer4) showing the best results in *in vitro* curated GIs also performed better in the other organisms analyzed. IslandViewer4 showed the highest percentage of possible true-positives (common GIs), followed by GIPSy, and Alien Hunter. Zisland Explorer presented intermediate results. GI Hunter and Predict Bias failed to predict 50% of the total results as possible true-positives.

Considering single islands, GI Hunter and Predict Bias failed to achieve satisfactory results, with percentages of possible false-positives of 51–67%, respectively. Zisland Explorer presented an intermediate result compared to the other tools. IslandViewer4 featured the lowest (13%) unique GIs, followed by GIPSy, and Alien Hunter.

As previously mentioned, when comparing the best results of the gold standard GIs with our dataset, we found that the tools showed similar performance. Thus, we recommend combining the three tools with better performance in this study to improve the results. Alien Hunter generates an overview of each predicted GI indicating if the regions contain ribosomal DNA sequences; if the genome is newly assembled, it can be analyzed without prior annotation. IslandViewer4 provides a more interactive and dynamic search of genes present in candidate GIs and information about possible genes associated with pathogenicity and antibiotic resistance. GIPSy complements the analysis because of its various integrated methodologies and ability to identify tRNAs and classify islands according to their possible functions.

## Author Contributions

AdSF, the lead author, generated the results and wrote the article. RR and JM contributed to the development of all processes. DG and DA contributed to the testing of the tools. CD aided in article writing. IdS-W reviewed the article.

### Conflict of Interest Statement

The authors declare that the research was conducted in the absence of any commercial or financial relationships that could be construed as a potential conflict of interest.
