# Supplementary material for: Comparative Analysis of Genomic Island Prediction Tools
Source: Front Genet. 2018 Dec 12;9:619. doi: 10.3389/fgene.2018.00619 (PMC6315130; doi:10.3389/fgene.2018.00619)
Supplement: Supplementary file 1 [file Table_1.DOCX]

Supplementary Material

ANALYSIS OF GENOMIC ISLAND PREDICTION TOOLS

Antonio Camilo da Silva Filho^1*^, Roberto Tadeu Raittz^1^, Dieval Guizelini^1^, Camilla Reginatto De Pierri^2^, Diônata Willian Augusto^1^, Izabella Castilhos Ribeiro dos Santos-Weiss^3^, Jeroniza Nunes Marcahukoski^1^

^1^Departament of Bioinformatics, Professional and Technical Education Sector, Federal University of Parana, Curitiba, Brazil.

^2^Department of Biochemistry and Molecular Biology, Federal University of Parana, Curitiba, Brazil.

^3^Department of Clinical Analysis, Federal University of Parana, Curitiba, Brazil.

*** Correspondence:**Corresponding Author
antonio.camilofilho@gmail.com

# Supplementary Figures and Tables

## Supplementary Figures


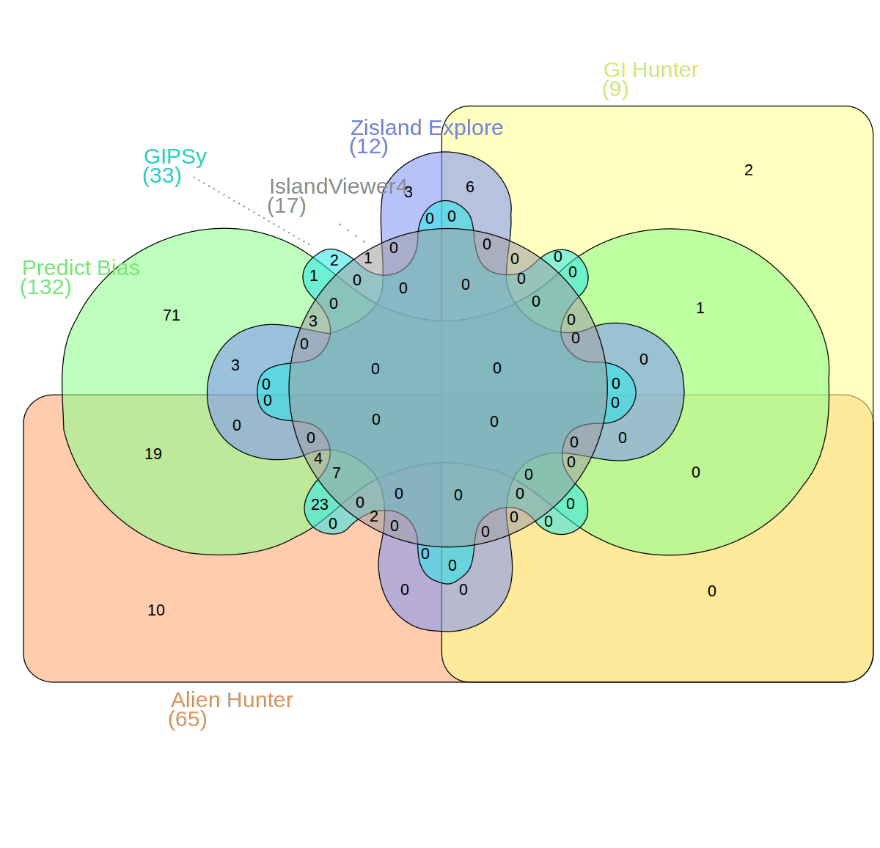


**Supplementary Figure 1.** Aeromonas hydrophila subsp. hydrophila ATCC 7966


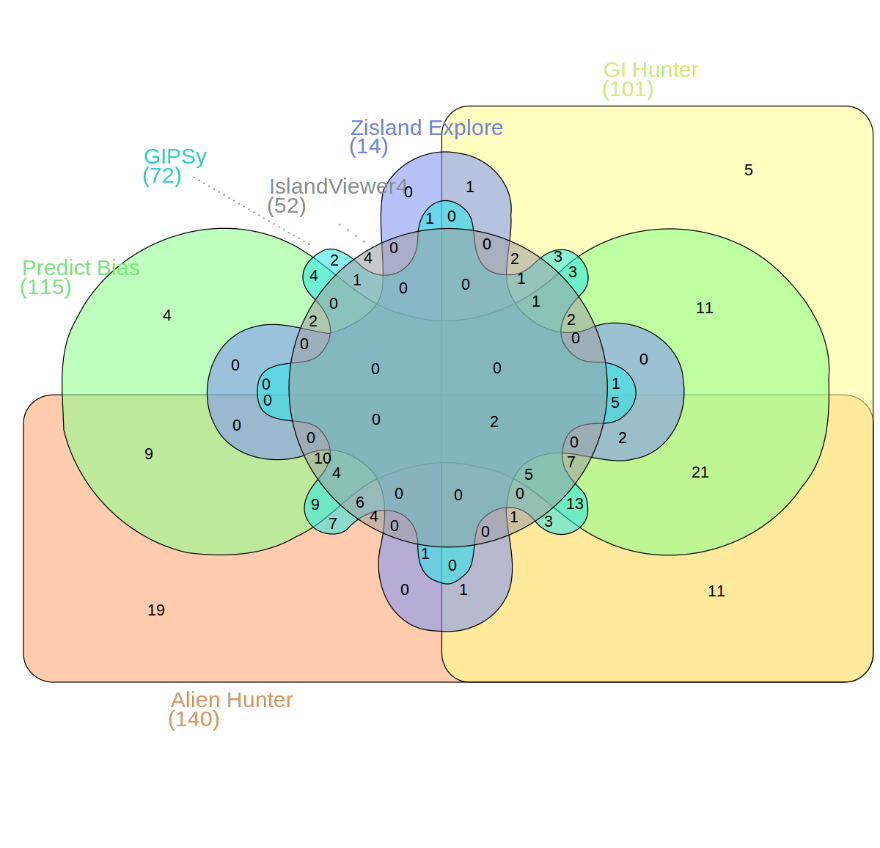


**Supplementary Figure 2.** Corynebacterium diphtheriae NCTC 13129


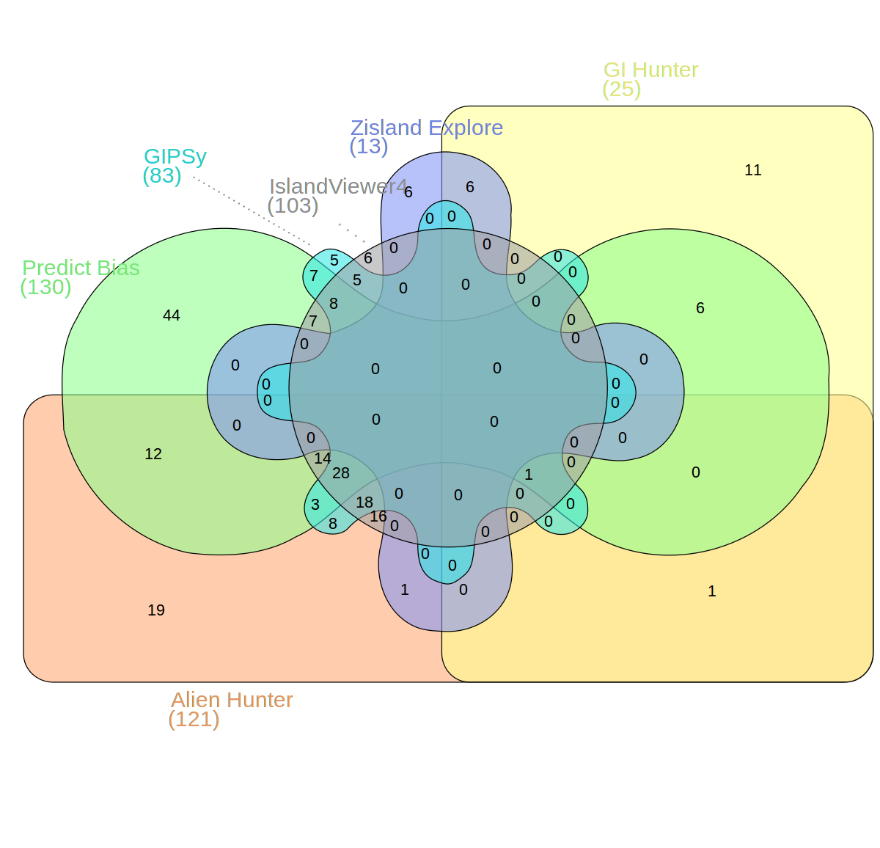


**Supplementary Figure 3.** Escherichia coli str. K-12 substr. MG1655


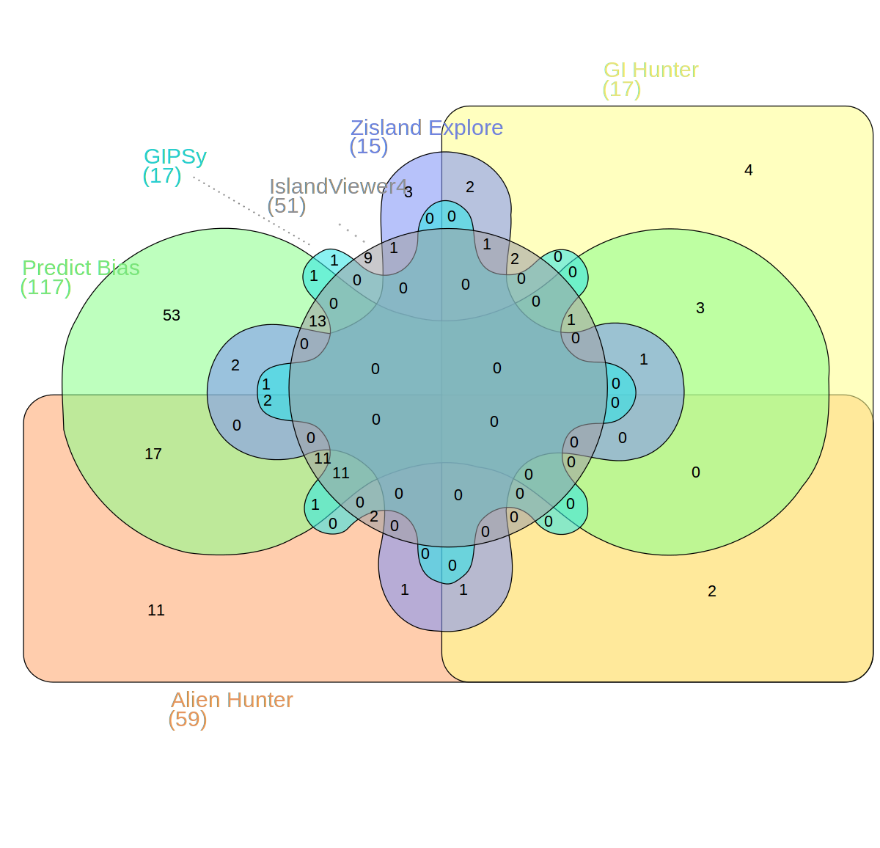


**Supplementary Figure 4.** Pseudomonas aeruginosa PAO1


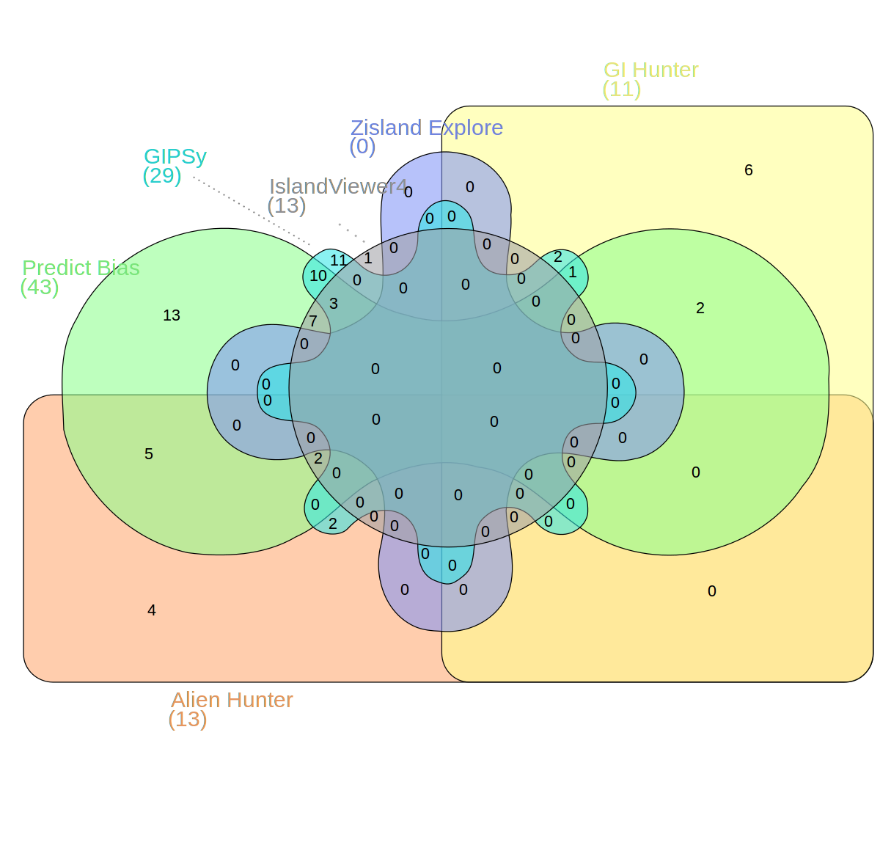


**Supplementary Figure 5.** Staphylococcus aureus subsp. aureus NCTC 8325


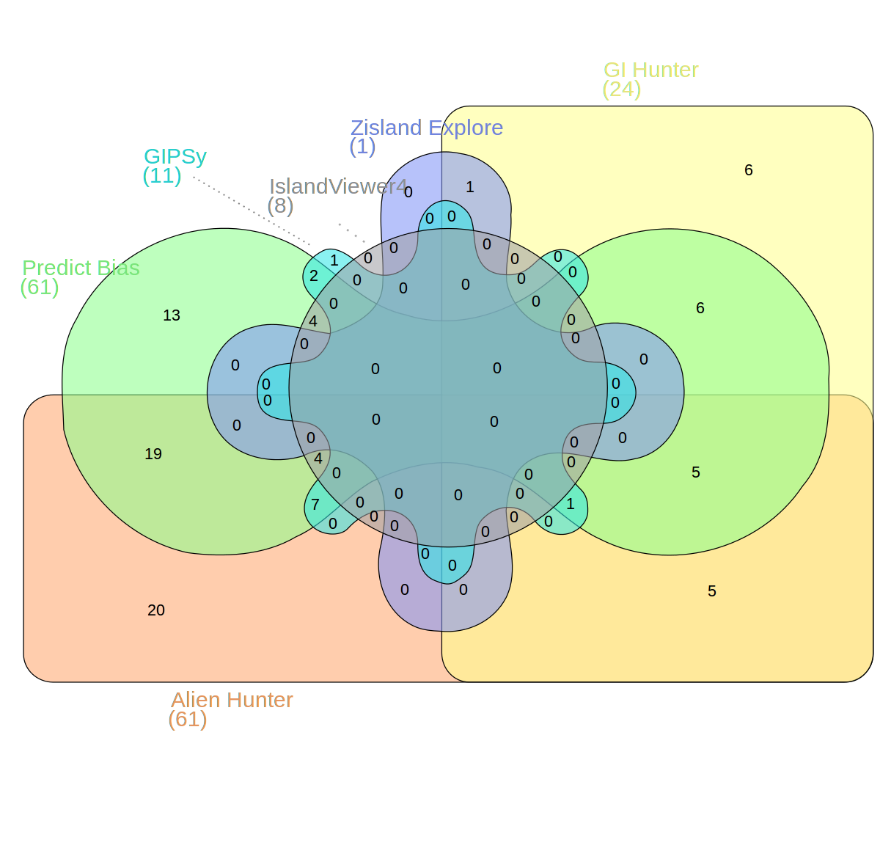


**Supplementary Figure 6.** Streptococcus pyogenes M1 GAS


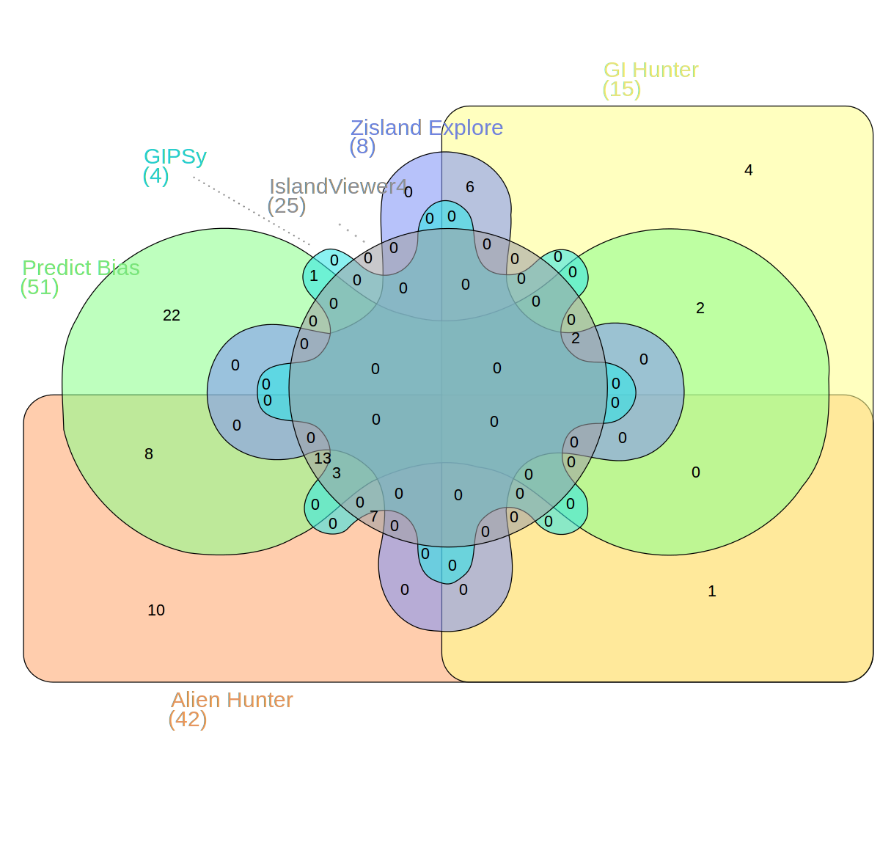


**Supplementary Figure 7.** Vibrio cholerae O1 biovar El Tor str. N16961 chromosome I


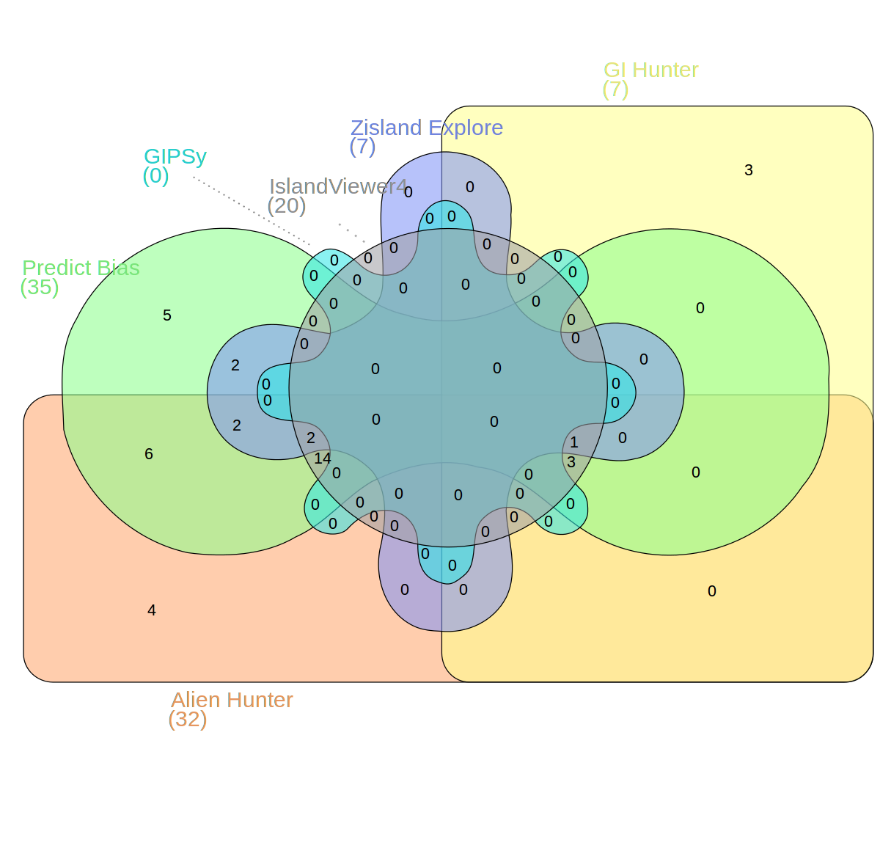


**Supplementary Figure 8.** Vibrio cholerae O1 biovar El Tor str. N16961 chromosome II


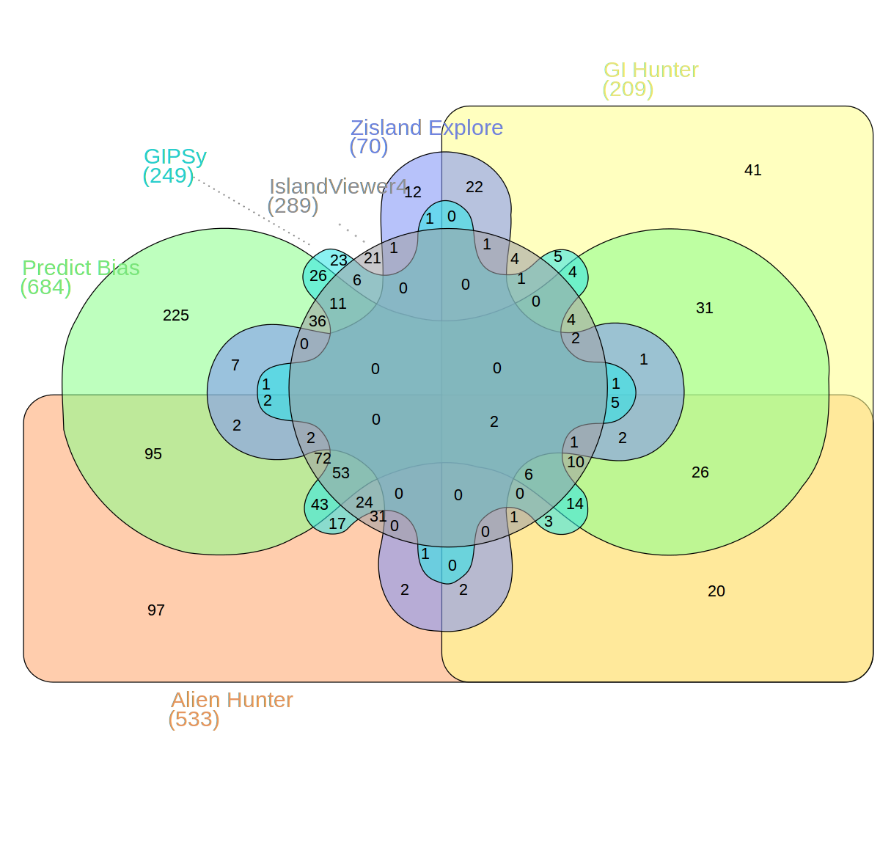


**Supplementary Figure 9.** Total number of hits between predictors in all organisms (Common GIs and Unique GIs). [Alien Hunter] (97); [GI Hunter] (41); [IslandViewer4] (21); [GIPSy] (23); [Predict Bias] (225); [Zisland Explore] (12); [IslandViewer4] and [Zisland Explore] (1); [Zisland Explore] and [GIPSy] (1); [IslandViewer4] and [GIPSy] (6); [Predict Bias] and [GIPSy] (26); [Alien Hunter] and [GIPSy] (17); [GI Hunter] and [GIPSy] (5); [GI Hunter] and [Predict Bias] (31); [Alien Hunter] and [GI Hunter] (20); [GI Hunter] and [IslandViewer4] (4); [Alien Hunter] and [IslandViewer4] (31); [IslandViewer4] and [Predict Bias] (36); [Alien Hunter] and [Predict Bias] (95); [Predict Bias] and [Zisland Explore] (7); [Alien Hunter], [GI Hunter] and [Predict Bias] (26); [Alien Hunter] and [Zisland Explore] (2); [Alien Hunter], [GI Hunter], and [Zisland Explore] (2); [Alien Hunter], [Zisland Explore], and [GIPSy] (1); [Alien Hunter], [Predict Bias], and [Zisland Explore] (2); [Alien Hunter], [GI Hunter], [Predict Bias], and [Zisland Explore] (2);[GI Hunter], [Predict Bias], and [Zisland Explore] (1); [Alien Hunter], [IslandViewer4], [Predict Bias], and [Zisland Explore] (2); [Alien Hunter], [GI Hunter], [IslandViewer4], [Predict Bias], and [Zisland Explore] (1); [GI Hunter], [IslandViewer4], [Predict Bias], and [Zisland Explore] (2); [Alien Hunter], [GI Hunter], and [GIPSy] (3); [Alien Hunter] and [Predict Bias] and [GIPSy] (43); [Alien Hunter] and [GI Hunter] and [Predict Bias] and [GIPSy] (14); [Alien Hunter], [IslandViewer4], [Predict Bias], and [GIPSy] (53); [Alien Hunter], [IslandViewer4], and [GIPSy] (24); [Alien Hunter], [GI Hunter], [IslandViewer4], [Predict Bias], and [GIPSy] (6); [Alien Hunter], [GI Hunter], [IslandViewer4], [Predict Bias], [Zisland Explore], and [GIPSy] (2); [Predict Bias], [Zisland Explore], and [GIPSy] (1); [Alien Hunter], [Predict Bias], [Zisland Explore], and [GIPSy] (2); [GI Hunter], [Predict Bias], [Zisland Explore], and [GIPSy] (1); [Alien Hunter], [GI Hunter], [Predict Bias], [Zisland Explore], and [GIPSy] (5); [GI Hunter], [IslandViewer4], and [GIPSy] (1); [GI Hunter], [Predict Bias], and [GIPSy] (4); [IslandViewer4], [Predict Bias], and [GIPSy] (11); [GI Hunter] and [Zisland Explore] (22); [GI Hunter], [IslandViewer4], and [Zisland Explore] (1); [Alien Hunter], [IslandViewer4], and [Predict Bias] (72); [GI Hunter], [IslandViewer4], and [Predict Bias] (4); [Alien Hunter], [GI Hunter], [IslandViewer4], and [Predict Bias] (10); [Alien Hunter], [GI Hunter], [IslandViewer4] (1).
